# Draft Genome Sequence of *Coxiella burnetii* Strain Cb196, an Agent of Endocarditis in Saudi Arabia

**DOI:** 10.1128/genomeA.01180-14

**Published:** 2014-11-26

**Authors:** Felicetta D’Amato, Catherine Robert, E. I. Azhar, Pierre-Edouard Fournier, Didier Raoult

**Affiliations:** a Aix-Marseille Université, Unité de Recherche sur les Maladies Infectieuses et Tropicales Emergentes, UM63, CNRS 7278, IRD 198, INSERM 1095, Marseille, France; b Special Infectious Agents Unit, King Fahd Medical Research Center, King Abdulaziz University, Jeddah, Saudi Arabia; c Department of Medical Laboratory Technology, Faculty of Applied Medical Sciences, King Abdulaziz University, Jeddah, Saudi Arabia

## Abstract

*Coxiella burnetii* Cb196, with a 2,006,415-bp genome, is a strain isolated from a 45-year-old man in Saudi Arabia with endocarditis. It belongs to the genotype MST51, which was detected for the first time only in this country. Cb196 shows more similarity to *C. burnetii* CbuK_Q154, belonging to genotype 8, which was phylogenetically close to MST51.

## GENOME ANNOUNCEMENT

*Coxiella burnetii* is a zoonotic and strictly intracellular Gram-negative bacterium belonging to the *Gammaproteobacteria*, and it is the agent of Q fever (1).

We isolated *C. burnetii* strain Cb196 from the cardiac valve, received in September 2012, of a 45-year-old man in Saudi Arabia with aortic endocarditis (2). Q fever endocarditis has been described in this country, both in children and adults (2–5).

Strain Cb196 was cultured in human embryonic lung cells using the shell vial method (6). Multispacer genotyping (MST), based on the variability of 10 intergenic sequences (7, 8), was performed and enabled the identification of a unique genotype, MST51 (2).

Sequencing was performed using the MiSeq technology with the paired-end and bar code strategies, according to the manufacturer’s instructions (Illumina, San Diego, CA, USA). Briefly, the library was mixed with 13 other genomic projects constructed according to the Nextera XT library kit (Illumina). The libraries were normalized and pooled into one single-strand library that was loaded onto the reagent cartridge and then onto the instrument along with the flow cell. Automated cluster generation and paired-end sequencing with dual-index reads were performed in a single 39-h run in 2 × 250-bp. A total output of 8.9 Gb was obtained with 1,023,000/mm^2^ density and 89.6% (19,294,000 clusters) of the clusters passing quality control filters. Within this pooled run, the index representation of Cb196 was determined to 1.31%. The 226,405 paired-end reads were filtered according to the read qualities. We followed the A5-miseq pipeline (9) for read trimming, correction, contig assembly, and scaffolding. We performed a further step of misassembling correction using the r2cat tool (10) and then reordered the scaffolds with Mauve (11) using Cbuk_Q154 (12) as the reference genome, having genotype 8 phylogenetically close to MST51.

Sequencing resulted in 3 scaffolds of 507,295 bp, 1,474,541 bp, and 24,579 bp (with 99, 100, and 99% coverage of the *C. burnetii* CbuK_Q154 genome, respectively). The genome size is 2,006,415 bp (G+C%, 42.9%), which is very similar to that of strain CbuK_Q154. The genome contains genes encoding 1,898 proteins, 44 tRNAs, and one ribosomal operon.

Compared to another 7 complete *C. burnetii* genomes, strain Cb196 had a similar gene function distribution. Strain Cb196, as CbuK_Q154, harbors the QpRS plasmid (39,312 bp).

In comparison with strain CbuK_Q154, strain Cb196 had 2,356 mutations, including 2,018 single-nucleotide polymorphisms (SNPs) (916 nonsynonymous among 1,358 SNPs in putative open reading frames [ORFs]), 171 insertions (57 in ORFs), and 167 deletions (63 in ORFs). A total of 674 genes were mutated. We classified the products of the nonsynonymous genes in COG categories. Forty-one proteins were classified in category J, 6 in K, 37 in L, 5 in D, 14 in V, 12 in T, 46 in M, 8 in U, 21 in O, 24 in C, 27 in G, 33 in E, 9 in F, 27 in H, 14 in I, 16 in P, 8 in Q, 58 in R, 42 in S, 34 belonged to multiple classes, and 192 were not annotated.

Statistical tests showed that for categories V and M, the proportions of mutated proteins were higher than for other protein categories (14/20 versus 660/1,878; bilateral chi-square test, *P* = 0.001; and 46/104 versus 628/1,794; *P* = 0.056, respectively).

### Nucleotide sequence accession numbers.

Strain Cb196_Saudi_Arabia has been deposited in GenBank under the project accession no. PRJEB7092, and the accession numbers are CCXO01000001 to CCXO01000003. The version described in this article is the first version.
